# An acquisition, curation and management workflow for sustainable, terabyte-scale marine image analysis

**DOI:** 10.1038/sdata.2018.181

**Published:** 2018-08-28

**Authors:** Timm Schoening, Kevin Köser, Jens Greinert

**Affiliations:** 1 GEOMAR Helmholtz-Center for Ocean Research Kiel, 24148 Kiel, Germany; 2 Christian-Albrechts University Kiel, Institute of Geosciences, 24118 Kiel, Germany

**Keywords:** Scientific data, Ocean sciences, Research data

## Abstract

Optical imaging is a common technique in ocean research. Diving robots, towed cameras, drop-cameras and TV-guided sampling gear: all produce image data of the underwater environment. Technological advances like 4K cameras, autonomous robots, high-capacity batteries and LED lighting now allow systematic optical monitoring at large spatial scale and shorter time but with increased data volume and velocity. Volume and velocity are further increased by growing fleets and emerging swarms of autonomous vehicles creating big data sets in parallel. This generates a need for automated data processing to harvest maximum information. Systematic data analysis benefits from calibrated, geo-referenced data with clear metadata description, particularly for machine vision and machine learning. Hence, the expensive data acquisition must be documented, data should be curated as soon as possible, backed up and made publicly available. Here, we present a workflow towards sustainable marine image analysis. We describe guidelines for data acquisition, curation and management and apply it to the use case of a multi-terabyte deep-sea data set acquired by an autonomous underwater vehicle.

## Introduction

Modern ocean science gear for underwater sampling is commonly equipped with optical imaging devices like photo and video cameras. These record valuable data for navigation, exploration and monitoring purposes. A multitude of strategies have been developed for various marine data acquisition and data management aspects. These include the design and deployment of underwater camera gear for scientific and industrial applications^1^, the curation and management of oceanographic data^2^, the acquisition of all data required for a full biological assessment of a habitat^3^ and references for manually annotating marine imagery^4^. Currently though, protocols are lacking for the steps following the marine image acquisition, namely these are: i) image data curation to quality control the recorded raw data and ii) image data management to publish the data sets in a sustainable way in work repositories and long-term data archives. Subsequent steps like manual image annotation and automated image analysis are even less standardized. Together this often leads to un-managed data in the form of dispersed copies on mobile hard disks which unnecessarily duplicate the data, prevent access controls and easily get lost or corrupted.

An additional need for more standardization exists due to the increasing popularity of autonomous underwater vehicles (AUVs). These can record large volumes of image data at an unprecedented acquisition velocity. AUVs are being deployed for large-scale assessments of the seafloor which require specific data processing workflows^5^. The trend towards parallel deployment of multiple AUVs will further increase the pressure in being able to efficiently curate and manage those big image data sets.

The scale of the image data management challenge is governed by the required image resolution and the area to be surveyed. An uncompressed, color ortho-photo of the entire seafloor, acquired at 1px/mm resolution, would require ca. 1 zettabyte of storage space (71%×5.10×10^8^×km^2^×3 bytes/mm^2^×1.09×10^21^ bytes). This is about 1/10th of all hard disk storage produced in 2017 and does not consider repeated monitoring for time series observations. Even a single imaging survey of 1 km^2^ seafloor coverage typically produces 0.5 TB of imagery.

Data management strategies that could address a challenge of this scale are rare in the literature. Related applications exist in other fields, e.g. in medical applications^6^ and concerning data provenance in big data sets^7^. Nevertheless, these strategies and applications cannot take into account the specific challenges in the data curation of marine data of which uncertain navigation and limited data transfer capability are the most obvious ones.

While some marine data archives have been set up, they are usually being used to publish one-dimensional or below-gigabyte data rather than hundred-thousands of high-resolution images. Furthermore, long-term accessibility is challenging to achieve. In a recent publication shallow water seafloor images-also acquired by an AUV-and manually created expert annotations for those images were published in an open access format^8^. While the annotations are still available in a long-term archive, the link to the imagery is already broken (http://data.aodn.org.au/IMOS/public/AUV/). This points out the need for long-term maintenance of data products and data archives-especially in times of global change when time-series studies of the natural environment need to be conducted and when scientific results are being questioned because of political motivation.

**Figure 1:**
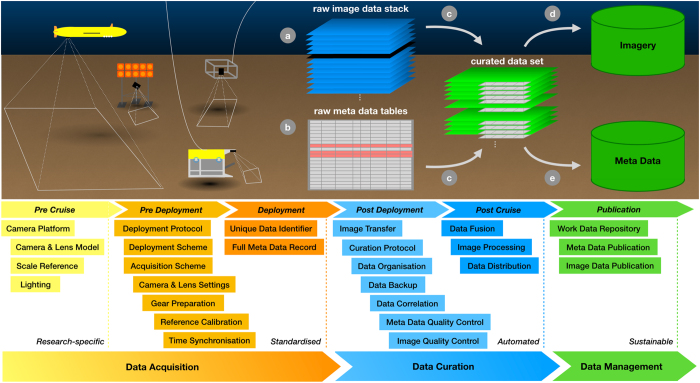
Schematic overview of the proposed image data workflow from acquisition through curation and management. Various robots (autonomous underwater vehicles (AUVs), landers, remotely operated vehicles (ROVs), towed platforms) create stacks of imagery (a) and metadata tables (b). Erroneous metadata values (here marked in red) and corrupt imagery (e.g. black images where the flash did not fire) might occur. Metadata are attached to the image data, image processing is applied and corrupt and erroneous data are flagged and filtered out (c). The resulting curated data set is the quality controlled data product that is suitable for publication and analysis. Metadata and image data are stored in suitable databases (public or private). Image data items should be linked to their corresponding metadata at archiving. The individual steps from pre-cruise planning to publication are discussed in the text. For a specific use case, see Fig. 3.

Here, we propose a marine image data acquisition, curation and management workflow (see Fig. 1). An AUV-based deep seafloor image data set is presented as a use case for the workflow. We elaborate the specific acquisition, curation and management steps for this use case in detail to explain the steps of the general workflow.

The image data of this use case, combined with its metadata and environmental data have been published in the long-term information system PANGAEA for earth and environmental science^9^, (Data Citation 1). This is the first time that a workflow for terabyte-scale deep-sea image data has been published and the first time that PANGAEA has been used for such large volumes of optical image data.

## Results

**Figure 2:**
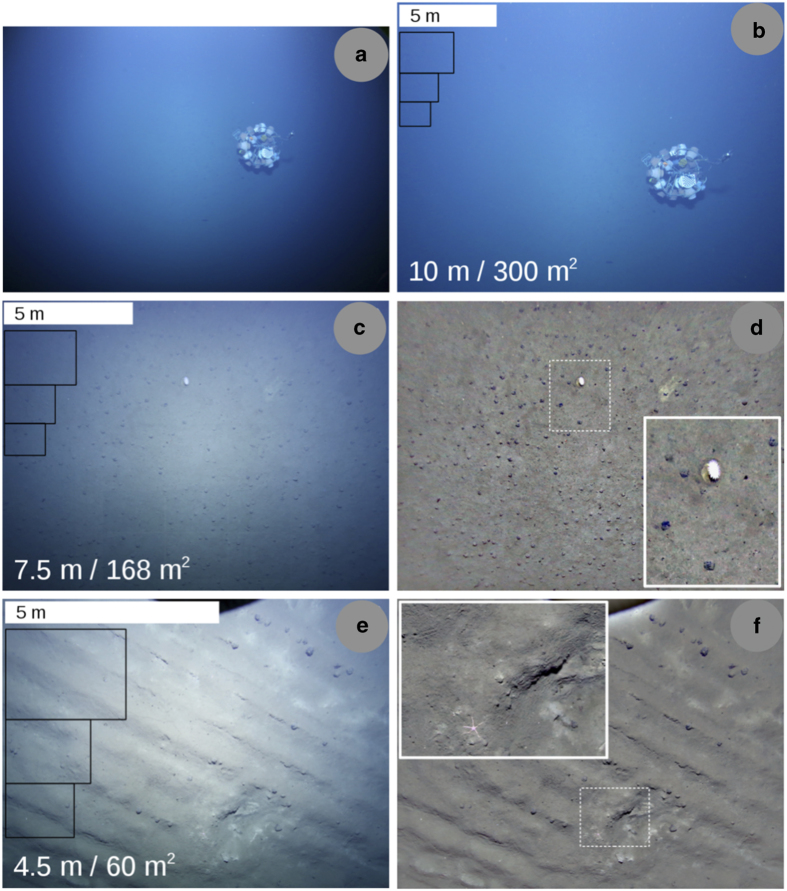
Example images from the presented use case image data set. Panel (**a**) shows a raw image taken by the camera onboard AUV Abyss from ca. 10 m altitude. The object in the middle is a stationary lander^26^ which was deployed independently of the AUV dives for environmental measurements. Panel (**b**) shows the effect of lens un-distortion. Black boxes show areas of 6, 3 and 2 m^2^ (top to bottom), corresponding to average footprints of other optical image acquisition gear (i.e. AUV, towed camera, ROV), computed for their usual operational altitude and field of view. Images in panels (**c**) and (**e**) are further un-distorted examples, taken at altitudes of 7.5 m and 4.5 m. Images in (**d**) and (**f**) are the results of a color normalization, applied to (**c**) and (**e**). Two zoom-ins (marked by the dashed, white box) show an anemone surrounded by poly-metallic nodules (**d**) and a sea star, close to a decades-old plow track that extend over the entire image (f, parallel linear structures).

**Figure 3:**
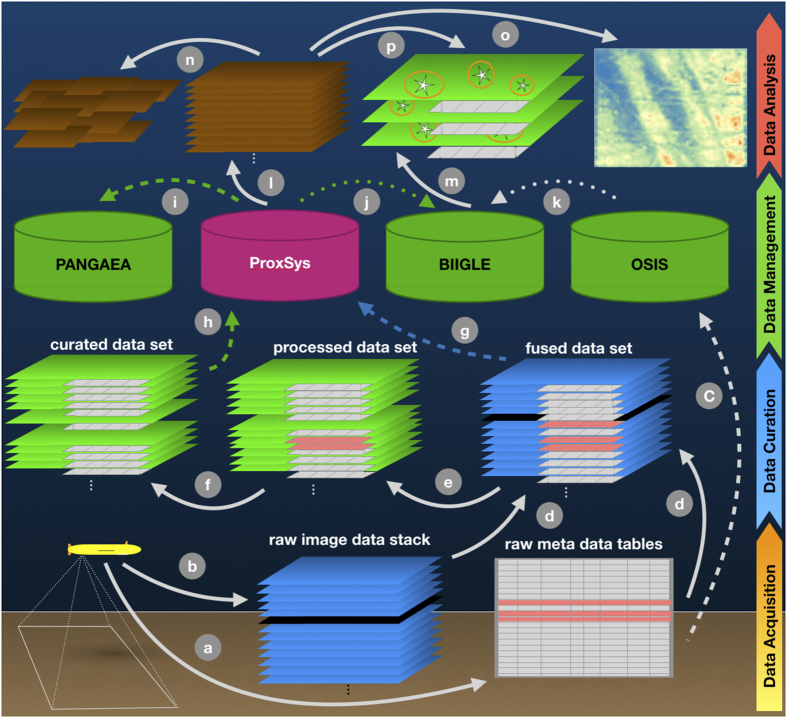
The data workflow as applied to the AUV use case. The AUV Abyss created metadata files (a) and stacks of up to 50,000 images (b) per dive. Meta- and image data were fused by time code (d). Un-distortion was applied (e), erroneous data were removed (f). Raw metadata are stored in OSIS (c). Raw and curated imagery is managed with ProxSys (g, h). Curated image data are made publicly available: in PANGAEA for long-term archival (i - by duplication) and in BIIGLE for manual annotation (j - by link). OSIS links to the image data in BIIGLE (k). Subsequent image analysis, enabled by the curated data are color normalization (i), mosaicking (n), mineral resource mapping (o), and automated event detection and classification within individual images (p), using manual annotations from BIIGLE (m) and machine learning.

Based on the use case described below, further experiences in digitizing decades old slides and managing large image data collections in-house and discussions within the international marine imaging community^10^, we propose the following steps to conduct sustainable marine data acquisition, curation and management for big marine image data sets (Fig. 1). A representation of the workflow steps with regard to the AUV image data set (see Fig. 2) use case is given in Fig. 3. Details of the data acquisition steps can be found in the literature^1^.

### Data Acquisition

Several decisions need to be made before a cruise, namely the selection of the camera platform, the camera system including lighting and a scale reference. These decisions are strongly dependent on the research-specific goal, thus no exhaustive review is possible here. Specific considerations are required prior to each individual deployment as well. Sensible decisions for a research-specific acquisition of data can reduce the amount of data to be recorded thus reducing the data volume challenge.

#### Camera Platform

Select a camera platform that allows a dive deployment (see below) to answer the scientific question. Large spatial mapping for example can be done by AUV, ultra-high-resolution imaging is better achieved by remotely operated vehicles (ROVs), stationary observatories can provide time-series observations.

#### Camera and Lens Model

Equip the camera platform with a camera system that resolves the objects of interest. The resolution in millimeters per pixel (mm/px) should be at least an order of magnitude larger than the size of the smallest object to be imaged.

#### Scale Reference

Use a camera system that provides scale information. This can come in the form of stereo cameras, a calibrated system with extrinsic data (position, orientation, altitude, etc.) or by laser pointers which provide reference scaling information directly embedded within the image data.

#### Lighting

Equip the camera platform with suitable lighting that is bright enough to illuminate the scene while not impairing the image acquisition by scattering. Low-light applications (e.g. for bioluminescence imaging) might work without lighting while further specific applications like coral imaging or non-impacting imaging might be conducted using ultra-violet (UV) or infra-red (IR) lights.

#### Deployment Protocol

Record all available information regarding the data acquisition to document the data provenance. This includes cruise information, deployment plan and derivations thereof, make and model of the camera, lens, lighting and optical port used. If camera characteristics are not available, at least the 35mm-equivalent focal length of the camera (providing the field of view in air) and the optical port type (dome or flat glass) must be recorded. A dive protocol is usually documented manually but should be digitized later on, stored alongside the imagery and ideally be published for future reference. To make the data as sustainable as possible, we additionally recommend photographing the entire capture system from multiple perspectives including a scale reference in order to allow a potential user to later check for details not yet considered.

#### Deployment Scheme

Select a dive scheme depending on the topography and suitable to answer the dive-specific scientific question: e.g. random, stationary, 1D transect, 1.5D mesh, 2D mosaic, 3D terrain. Systematic deployments should be conducted without stopping, zooming, panning, tilting and sampling.

#### Acquisition Scheme

Select the frequency of image acquisition. To enable photogrammetric reconstructions, large image overlap is required. This can come in the form of fast acquisition of still images or even videos. Other applications require quasi-random acquisition to prevent faunal adaptation, e.g. to the attraction of food by the light source. To prevent repeated counting of objects for systematic spatial analyses, the acquisition scheme could be designed to prevent image overlap. Otherwise, non-overlapping imagery would have to be selected in a post-processing step through cropping, tiling or filtering.

#### Camera and Lens Settings

Adjust the acquisition settings to the selected deployment and acquisition scheme. Fast acquisition rate increases data volume and might require a reduction in image resolution. Lens settings need to be adjusted to the available light.

#### Gear Preparation

Prevent moisture within the camera housing. It can be filled with dry air, cooled air or an inert gas. Additional drying agents should be contained and checked prior to each dive.

#### Reference Calibration

For precise measurements, e.g. in case of photogrammetric applications, repeated calibration of the camera setup is required. This can come in the form of checking stereo camera pair distances or laser distances but is usually conducted by taking calibration pictures. Typically, a calibration target like a checkerboard pattern is photographed from multiple view points.

#### Time Synchronization

Synchronize all clocks of all sensors. This should ideally be achieved by a technical solution like network time protocol (NTP), e.g. (ref. 11), and for synchronous capture (electrical) trigger signals are recommended (e.g. for stereo cameras).

#### Unique Data Identifier

Use an image naming scheme that encodes the cruise and station (including camera identifier, see Table 1) as well as image acquisition time in UTC (up to milliseconds) in each file name. Use a machine-readable format (numbers, characters, dashes and underscores only). A folder structure should be used (see below) but only to structure the data, not to encode further information.

#### Full Metadata Record

Record all required information to geo-reference each individual pixel. For geo-referencing objects seen in the images the camera's pose (position and orientation) in the world must be known at the time of each photo. Typically, information from ultra-short base line (USBL), Doppler velocity log (DVL) and inertial measurement unit (IMU) is fused to determine the pose of the platform where the camera housing is mounted. This pose refers to the platform's reference point and axes (the vehicle coordinate system). It is important to measure the position and orientation of the camera with respect to the vehicle coordinate system. We recommend storing the position as a 3-vector in meters, and the rotation as a 3-by-3 rotation matrix that takes a direction vector in camera coordinates and computes the same direction in world coordinates. Such information should be stored for every camera, light and possibly other sensors of the system. Standardized fields, based on the PANGAEA archive should be used to ease data interchange between image analysis softwares (see Table 1). Additionally, record all available environmental and further metadata that are related to the image acquisition.

### Data Curation

Data curation, especially quality control and documentation steps should happen as soon as possible after acquisition to prevent knowledge loss.

#### Image Transfer

The data transfer needs to be adjusted to the cruise plan and the data analysis requirements. For immediate use of imagery, the fastest data transfer is required to feed the data into the processing computer. This is usually achieved by mechanically extracting the storage device. A less laborious way to speed up the transfer is to use lossless data compression during acquisition to reduce data transfer overheads for small file sizes and large file numbers.

#### Curation Protocol

Complementary to the deployment protocol, the data curation also needs to be documented. As these steps are already mostly automated-and ideally fully automated in the near future-digital documentation is recommended. By combining the documentation with the actual processing tools, this can come in the form of a re-usable dynamic protocol, e.g. using Jupyter notebooks^12^.

#### Data Organization

Large volumes of imagery need to be split up in a meaningful and effective manner. Individual folders should contain less than 1,000 image files. Splitting limits could be set per index, per time, per distance travelled, per data volume or something else. Folders need to have meaningful names to aid data discovery but should not uniquely encode acquisition metadata.

#### Data Backup

Both image and metadata needs to be duplicated as soon as possible to prevent data loss. Even on research vessels those copies should be physically separated to prevent destruction by water, fire, mechanical trauma etc.

#### Data Correlation

Perform data-driven sensor checks by cross-correlating data series of separate sensors to ensure internal consistency of fused data sets.

#### Metadata Quality Control

Perform quality control of all metadata. This includes correcting for time offsets, removal of errors and outliers and interpolation of missing values. When noisy data are smoothed, the dive protocol needs to include the smoothing procedure that has been applied to enable re-use and to maintain data provenance.

#### Image Quality Control

Check image quality to flag or filter out corrupt images (e.g. all black, obstructed view, condensation, overexposure, turbidity). Some image quality control can be conducted automatically using data-driven heuristics, others need manual inspection.

#### Data Fusion

Store metadata alongside the imagery. This can come in the form of a physical fusion of data in a single file, by storing metadata in a separate ASCII text file next to the imagery in the image archive or by setting up a separate repository (e.g. Git) for the metadata files. A repository is favorable as it efficiently allows for changes of the metadata without the need to change the image data while maintaining data provenance. Anyhow, these are not mutually exclusive: e.g. a combination of a physical fusion into the file header and a separate repository can be used together.

#### Image Processing

The imagery to be published should be of a quality that enables immediate analysis. In some cases this might require an image preprocessing step (e.g. RAW conversion, lens un-distortion, color spectrum normalization). If image processing is applied to construct the curated image data set, the processing algorithms and parameters need to be recorded alongside the constructed data set to maintain data provenance information.

#### Data Distribution

Upon return from an image acquisition campaign, copies of the entire meta- and image data should be geographically distributed. This can come in the form of mobile hard disks for participating researchers, institute-based storage infrastructure or cloud-based storage.

### Data Management

Once data have been curated, it needs to be made accessible for repeated use and long-term archival.

#### Work Data Repository

A work repository is beneficial when data are to be stored in a central location and to be used frequently by several individuals. Such repositories are already in place in many research institutions. In order to streamline the data curation and management process those repositories should feature application programming interfaces (APIs) to ease data import and export.

#### Metadata Publication

Metadata should be published immediately to foster citation and reuse of existing data sets.

#### Image Data Publication

Make curated image data available online and assign a digital object identifier (DOI). This should happen as soon as possible to support data-derived products and to make those products more transparent (scientific papers, reports, management/governing decisions). Assigning DOIs allows data provenance to be documented in subsequent analysis steps and provides acknowledgement by enabling attribution of curation and management efforts.

## Discussion

While the creation of the workflow was guided by mobile benthic imaging, it is similarly applicable to related scenarios like pelagic imaging and time-series observation. Indeed, it has already been applied to further, non-benthic imagery.

Another related, yet slightly different challenge that was largely neglected in this publication, is the curation and management of video data. Most of the curation and management steps presented here apply to videos as well but there are some significant differences. Data volume is usually an order magnitude higher compared to imagery and processing algorithms tend to be slower. The increased volume will slow down the data transfer which is sometimes compensated by recording in a lower resolution in parallel and first transferring the low-resolution data. The full metadata record needs to include frame information (e.g. frames per second). Image quality control cannot remove corrupt frames as this would corrupt the entire video without meaningful compensation. Data fusion for the file-header-based approach is not possible the same way as for imagery due to the different header structure and the necessity to store temporal metadata: values are required for each frame of the video which can be too much data for a file header. Software tools to browse and annotate video data will be different from the tools presented here. The presented workflow is thus not directly applicable to video without modifications. However, when cutting videos into separate frames, the workflow can be applied directly.

Depending on the application scenario it may be necessary to acquire imagery in raw format with higher dynamic range. Especially in low light situations, this allows better color corrections exploiting the increased bit depths. With regard to a sustainable curation of such imagery a further processing step would be necessary. This step would transform the raw imagery to a file format that can be displayed on most computers and thus be used for further interpretation. This transformation step would in turn increase the time needed for data curation and multiply storage requirements.

Standardization and an immediate curation and management at sea might reduce the data dispersal on singular hard disks that are shared during and after cruises. Those disks are often shared on a per-person basis, can thus contain data at various stages within the curation workflow and hence make comparability and data provenance monitoring challenging.

To maintain data provenance and to keep track of potential changes in data when novel curation procedures are employed it is necessary to link the long-term archived data with repositories that can document those changes. In the case of software, snapshots (e.g. released versions) should be published as static archives with a reference to the source code repository that is used to develop the software further (e.g. ref. 13). The same applies for metadata repositories where static snapshots are published as the current-state-of-the-art of the data product. This snapshot can always be referenced by a DOI in the future. The DOI handle would further refer to subsequently added versions of the data product that might have been created by methodology updates or to correct previously unknown errors.

The image and metadata set of the use case described in the Methods section is the first application case for the proposed image curation and management workflow towards sustainable marine image data publication. Many steps of the workflow have not yet been optimized for fast execution. Managing the large amounts of image data is a new and challenging task during research cruises. It requires fast computers, effective handling of the files as well as efficient algorithms to process the images in sufficiently short time. The time needed to copy images across hard disk drives (HDDs) and network attached storages (NASs) is unavoidable and slows down the data analysis process, especially in the first hours after a dive when scientists wait to see the unseen seafloor.

To speed up this step the file size of image archives can be tuned to increase the transfer rate. For the AUV use case, this size was chosen heuristically but depends on the CPU power of the compressing/decompressing computer, the file system and the packet size. A cruise-specific tuning of the archive size could further speed up the data transfer (adapted to the available interfaces, cruise schedule, immediate data analysis requirements, etc.).

Several of the manual steps can be automated in the future to further speed up the workflow execution. This would allow a further reduction in the AUV turnaround time and could speed-up subsequent scientific interpretation of the data. For AUV Abyss, the operational limit to the turnaround time is the exchange of batteries (ca. 3 h from recovery to re-deployment) while the digital limit is the download of the data which could take up to 9 h (ca. 2 h for common deployments). Additional image selection strategies, automated pre-clustering and visual overview displays^14,15^ should be implemented in the future to speed up the subsequent semi-automated exploration of data.

Currently, massively-parallel image analysis compute clusters are being built specifically for at-sea deployment. These would benefit from standardized data as provided by the workflow by removing the need to adapt the data analysis algorithms to each new data set with different parameters and data formats. Thus such clusters will enable terabyte-scale offshore image analysis.

Workflow steps might change over time with newly emerging technologies. The AUV use case will in the future likely employ the GEOMAR workbench rather than the OFOP software to fuse metadata files (available at https://dsm.geomar.de). Also the blockchain technology^16^ will be explored to be implemented as a mechanism to monitor data provenance.

A full standardization of the image and metadata workflow cannot be achieved. This is due to varying institute policies and tool availability. Meaningful software elements specific to curation and management steps will depend on the choices made during data acquisition. Necessary metadata fields as proposed in Table 1 can depend on each other (e.g. for dome port housings other CAM_alignment data are needed than in the case of flat ports). The workflow is presented here in a general form to describe the framework of tasks and how they are interrelated. It is also presented in a specific use-case to outline choices for tools and to discuss challenges. Together it should be possible to use the framework to provide curated marine image data for other stakeholders that can be exchanged and can efficiently be accessed by future users.

## Methods

Image and metadata for the use case were acquired during two expeditions of the German research vessel Sonne (SO239 (ref. 17) and SO242/1 (ref. 18)). The cruises targeted areas in the Pacific Ocean that were subject to simulated deep-sea poly-metallic nodule mining activities in past decades. Those nodules are a mineral resource, lying embedded in the sediment on the seafloor^19^. Mining is governed by the International Seabed Authority for areas that lie outside national waters. This UN organisation provides countries with license areas to conduct resource exploration. Four license areas within the Clarion-Clipperton Fracture Zone were targeted during cruise SO239 (German license area, Inter Ocean Metal license area, Belgian license area, French license area) and one Area of Particular Environmental Interest^20^. During SO242/1 the main DISCOL Experimental Area (DEA)^21,22^ was targeted as well as reference areas in the vicinity (<6 km). The specific research objective was to map poly-metallic nodule occurrence over tens of hectares. Quantitative results of naturally occurring heterogeneity were needed as predictors for faunal abundance and for mining related objectives.

### Data acquisition

GEOMAR's REMUS 6000 AUV Abyss was deployed as a camera platform to achieve the research objectives^23^. During cruise SO239, the Deep Survey Camera (DSC) system was used for the first time^24^. It employs a Canon 6D DSLR camera and a 15mm fisheye objective lens. The ground resolution of the fisheye image drops from the center of the image towards the boundaries. At 10 m altitude it is roughly 0.2 px/mm in the image center and is approximately doubled at the lowest flying altitude of 4.5 m. This means that structures of ca. 10 mm size can be resolved at 10 m altitude (ca. 5 mm at 4.5 m altitude). The camera system was calibrated in air using a checkerboard. Scale reference was thus provided using the calibration data and the altitude data of the AUV. Lighting is provided by custom-built LEDs (320,000 lumen) to enable imaging from high altitudes^25^. The lighting of the LEDs is the only illumination in the deep-sea and is flashed for 4 ms. The shutter speed of the camera has no effect as long as it is slower than the flash time and synchronized with the LEDs.

As the AUV is a torpedo-shaped vehicle that has to fly at a minimum speed, detailed terrain models are required to avoid collisions and the AUV has to keep a safe distance from the ground. Characteristics of most of the dive sites had been collected on earlier cruises, using still and video cameras, but these provided only geographically isolated snapshots. Hence, a mow-the-lawn deployment scheme was used to create contiguous 2D mosaics of large areas. For some dive areas, no previous terrain data were available and hence 1D transect deployments were chosen. That way an even larger area was covered but without overlap between the dive tracks, preventing the creation of mosaics. Detailed dive information is available in the cruise reports^17,18^.

The AUV flies at a speed of 1.5 m/s and the DSC was programmed to acquire images at 0.5–1 Hz. Depending on the altitude above the seafloor, an image overlap of up to 90% was created along track. Additional overlap across track was introduced by adjacent track lines spaced as closely as 3 m.

The DSC operated at fixed shutter and aperture settings. As autofocus does not work in our setting, focus and aperture have to be preset to a useful range before the dive. ISO speed was automatically tuned to the albedo of the terrain and the altitude as discussed in ref. 24.

The camera pressure housing was stored in a cold lab (ca. 4 °C) for a minimum of 30 min prior to each dive and closed inside to minimize condensation issues at cold water temperatures. Before each dive, the clocks of the AUV navigation computer and the camera computer were manually synchronized.

Custom-built camera operation software, based on the Canon camera software development kit (SDK), was implemented to record the images. Image file names were set to the pattern:

$$<\mathrm{cruise}\;\mathrm{name}\;>\;{}_{-}\;<\mathrm{station}\;\mathrm{number}\;>\;{}_{-}\;<\mathrm{date}:\;\mathrm{YYYYMMDD}\;>\;{}_{-}\;<\mathrm{time}:\;\mathrm{hhmmss}\;>\;{}_{-}\;<\mathrm{index}\;>$$

e.g.: SO239_115_AUV9_20150407_175001_IMG_0001). Images were stored as JPEGs to to preserve hard disk space and maintain a high acquisition rate.

As metadata, AUV Abyss provides navigation computed using long base line (LBL) beacons and its built-in Acoustic Doppler Current Profiler (ADCP), attitude and vehicle state data as well as conductivity, temperature, depth (CTD) and environmental data (e.g. turbidity, chlorophyll concentration).

21 dives were conducted in total, yielding 469,967 images (ca. 3.4 TB with lossy JPG compression at a factor of 98, ca. 30 TB uncompressed). A value of ca. 1 EUR per image was estimated, including all attributable costs (ship-time used, personnel hours, equipment used, etc.). During the first missions, AUV Abyss flew at an altitude of 12 m above the seafloor as a safety precaution. Later it operated at 7.5 m altitude and for the last four dives of cruise SO242/1, altitudes of 6 to 4.5 m were flown. Due to the illumination cones of the LED flashes, the light attenuation under water and the objective lens characteristics, the images show an illumination drop-off towards the corners (see Fig. 2 (a)). The effect of the altitude can be seen by comparing Fig. 2, panels (a),(c),(e). The adaptive ISO setting mostly created ISO speeds of 6,400 at ca. 320,000 lumen light intensity. The maximum possible ISO of the camera is 102,400.

### Data Curation

After each AUV dive in camera configuration the images were downloaded from the hard disk in the pressure housing on board Abyss to a mobile hard disk drive. Depending on the subsequent dive schedule, the transfer was conducted via Ethernet or by disassembling the camera pressure housing and retrieving the camera hard disk. Retrieving the hard disk is a laborious task that takes ca. 0.5 h but speeds up the data downloading due to the higher SATA/USB transfer rates. More efficient data transfer was achieved by pooling images in uncompressed file archives of 50 GB size. Although overheads for archiving and un-archiving are introduced, the overall transfer time was reduced because of reduced overheads for the Ethernet/USB transfer.

After extraction of the images from the archive, the data were split into sub folders, containing half an hour of images each (1,800–3,600 images). This step was necessary as even modern operating systems have difficulties in browsing and displaying folders containing more than a few thousand files.

Raw imagery and metadata were triplicated on three NASs for backup. One NAS served as the working repository to distribute data on the ship.

Metadata and imagery were acquired by separate recording systems. A data-driven strategy was implemented to compute the static millisecond time offset between these two systems. First, the average brightness of each image was computed as the average pixel gray value intensity of the main diagonal of an image. As high brightness is expected for low altitude, these two data series (image brightness, AUV altitude) were cross-anti-correlated to determine the best fitting time offset. As image acquisition times were recorded with millisecond accuracy and metadata values at seconds only, metadata had to be interpolated to be matched to the images. A linear interpolation was used and the entire cross-correlation was implemented in C++ for computational speedup.

Navigation and environmental metadata files were quality-controlled with custom-built PHP scripts, to find outlier values and empty data points. Afterwards the different metadata files (for navigation, environment, etc.) were merged by timecode using the Ocean Floor Observation Protocol (OFOP) software^27^. Missing data values were reconstructed through spline interpolation using OFOP.

During some dives, images were acquired in the water column in the ascent and/or descent phases. The AUV altitude sensor was used to automatically filter out all images acquired at altitudes above 10 m above the seafloor. Additional automatic filtering was applied to remove any images with impaired illumination by removing images of a mean gray value intensity below a manually chosen threshold to exclude images too dark for analysis. Condensation occurred at the camera dome port sometimes, despite cooling and drying the air inside the camera pressure housing. Image subsequences showing condensation were manually removed.

Selected values of the curated metadata were fused with the imagery by adding it to the EXIF header. This step was conducted with the software ExifTool^28^. Alongside author and copyright information, the latitude, longitude, altitude above seafloor and AUV heading were stored within the images. This complements the existing EXIF data on camera and lens models and settings. Together, this information allows geo-referencing each pixel in each image individually up to the accuracy of the AUV navigation data.

Fisheye lens un-distortion was conducted as rectilinear images are easier to process and analyze. The raw images were un-distorted to virtual images that an ideal perspective camera with 90° horizontal field of view would have seen from the same position. Therefor the color of each pixel in the ideal image is obtained by 1) computing the ray in space associated with this virtual pixel (using rectilinear un-projection), 2) projecting this ray into the raw wide angle image (using equidistant projection), yielding a sub-pixel position and 3) interpolating the colors of the neighboring pixels. Technically, the un-distortion has been performed using the tool biasproject from the Basic Image AlgorithmS Library. Metadata were retained within the processed images.

Alongside the raw images and metadata the curated metadata and imagery were also triplicated on the NASs. To prevent data loss through baggage loss or disaster, the NASs were split up after the cruises between different flights and containers. Three of the participating institutions (Senckenberg, GEOMAR, Bielefeld University) received one copy of the data each.

The image data curation led to the removal of 116,006 images (SO239: 62,948; SO242/1: 53,058) and left 353,961 images for publication and processing. For each of these images, navigation and environmental metadata were available, of which selected values were written to the EXIF file header.

Massive time offsets between the image acquisition time and the metadata acquisition time were observed initially during at-sea metadata curation. These offsets were due to human error and immediately corrected. Later time offsets ranged between tens of milliseconds to tens of seconds.

For all images acquired during cruises SO239 and SO242/1, 39.4 days of processing were required for the various data curation steps (single core timing, executed intermittently over a longer time frame and partly in parallel on a 3.5 GHz Hex-Core computer with 64 GB RAM). Examples of the curated images are given in Fig. 2(b).

### Data Management

After the cruise, the raw and undistorted images were stored in the GEOMAR media repository ProxSys (https://www.teltec.de/proxsys/) for in-house analysis. ProxSys allows for a versioning of the imagery. The original images were checked into the repository first and constitute version 1.0. Afterwards the curated images were checked in as version 1.1.

Metadata and cruise information were made available publicly using the Ocean Science Information System (OSIS, https://portal.geomar.de/osis).

As ProxSys is restricted to in-house use, all curated images were made publicly available through the annotation software BIIGLE 2.0 (ref. 29). BIIGLE provides an interactive working environment for sustainable and robust image annotation with quality-control mechanisms and annotator bias reporting. It is the state-of-the art software for marine image annotation. The DIAS prototype of BIIGLE 2.0 was operated at sea to gather annotations during the cruises. The annotation database created onboard RV Sonne was later transferred to the BIIGLE instance at GEOMAR (https://annotate.geomar.de).

All curated images were further transferred to the world data center PANGAEA^9^ for long-term archival. Each AUV dive was uploaded as a distinct data set (Data Citation 1). No embargo was installed and the curated images made publicly available immediately after uploading.

Based on curated data, further image processing and analysis steps were conducted for various scientific purposes. A detailed description of the image processing is out of the scope of this paper, but they are briefly described here as examples for users of well-curated and well-managed image data.

Geometric image analysis was conducted by computing multi-hectare mosaics using software under development. Suitable dives were selected where an AUV dive pattern with sufficient image overlap had been conducted. As the mosaics are geo-referenced, they allow spatial analyses at centimeter to 100m scale. Faunal characteristics can be analyzed over space, time and pixel-resolution by comparison to imagery acquired by ROVs and towed cameras in past decades.

In parallel, semantic image analysis was conducted to derive quantitative data on poly-metallic nodule occurrence. The Compact Morphology-based Nodule Delineation (CoMoNoD) algorithm was used for this task^30^. It employs a contrast-enhancing image processing to ease nodule segmentation from the sediment background. Afterwards, each individual nodule is delineated, its size measured and size statistics computed for subsequent geological interpretation. The source code for the nodule quantification has been published in PANGAEA^13^ and the detection results are available as well (Data Citation 2).

High-resolution nodule occurrence maps were computed to assess spatial patterns at meter-scale. Therefore, images were gridded to 1 m^2^ tiles and each tile was geo-referenced using the curated metadata available in the file header (latitude, longitude, altitude, heading). This nodule data are currently being used for biological, geological^31^ and information-theoretical studies.

## Usage Notes

Raw images are available on request and feature a wider field of view, which results in a dark image boundary due to the LED illumination drop-off towards the outer sectors.

## Additional information

**How to cite this article**: Schoening, T. *et al*. An acquisition, curation and management workflow for sustainable, terabyte-scale marine image analysis. *Sci. Data* 5:180181 doi: 10.1038/sdata.2018.181 (2018).

**Publisher’s note**: Springer Nature remains neutral with regard to jurisdictional claims in published maps and institutional affiliations.

## Figures and Tables

**Table 1:** Metadata fields to be stored alongside each image to geo-reference each pixel of an image.

**Tag**	**Description**
SUB_datetime	A date-time-stamp in the format "YYYYMMDD hh:mm:ss.sss"
SUB_latitude	Latitude position of the camera platform in decimal degrees
SUB_longitude	Longitude position of the camera platform in decimal degrees
SUB_distance	Altitude of the camera platform above ground
SUB_heading	Direction of travel along the x-axis, 0=North, 90=East
SUB_forwardvelocity	Speed of the camera platform along the x-axis
SUB_yawangle	Yaw angle (rotation around z-axis, see^1^)
SUB_pitchangle	Pitch angle (rotation around y-axis, see^1^)
SUB_rollangle	Roll angle (rotation around x-axis, see^1^)
CAM_model	Manufacturer and type of camera
CAM_id	Machine-readable camera identifier for multi-camera systems
CAM_position	Relative offsets of camera center in camera platform coordinates
CAM_orientation	Relative orientation of the camera to the camera platform
CAM_refraction	Refraction data: glass port type, glass thickness, refractive index
CAM_alignment	Port offset and normal in camera coordinates
CAM_lensmodel	Manufacturer and type of objective lens
CAM_focallength	Focal length in mm
CAM_fnumber	Objective lens aperture information
ENV_temperaturewater	Optional temperature parameter
ENV_absorption	Optional parameter for the absorption coefficients of the water
ENV_scattering	Optional parameter for the volume scattering function
ENV_refractiveindex	Optional refractive index parameter of the water around the camera platform
REF_laserdistances	Optional parameter if laser points are used for scaling
These represent the best-case scenario where all parameters are easily measurable. We propose to use these exact tag terms to enable data interchange between image analysis softwares. The chosen tags are derived from the field names used in the world data center PANGAEA for arbitrary marine data (changed to lowercase and without blanks and special characters to streamline automated processing). All lengths measurements in mm.	
